# *COCH*-Related Hearing Loss in a French Cohort: Novel Variants and Genotype–Phenotype Correlations

**DOI:** 10.3390/genes17050588

**Published:** 2026-05-21

**Authors:** Ralyath Balogoun, Margaux Serey-Gaut, Véronique Pingault, Isabelle Lemiere, Geneviève Lina-Granade, Geoffroy Delplancq, Anne Marie Guerrot, Annick Toutain, Delphine Dupin-Deguine, Marine Legendre, Estelle Colin, Natalie Loundon, Laurence Jonard, Sandrine Marlin

**Affiliations:** 1 Department of Genomic Medicine for Rare Diseases, Necker–Enfants Malades Hospital, Assistance Publique-Hôpitaux de Paris (AP-HP), 75015 Paris, France; margaux.sereygaut@aphp.fr (M.S.-G.); veronique.pingault@inserm.fr (V.P.); isabelle.lemiere@aphp.fr (I.L.); laurence.jonard@aphp.fr (L.J.); sandrine.marlin@aphp.fr (S.M.); 2 National Reference Center for Genetic Hearing Loss, Necker–Enfants Malades Hospital, Assistance Publique–Hôpitaux de Paris (AP-HP), 75015 Paris, France; 3 Department of Otorhinolaryngology and Audiophonology, Edouard Herriot Hospital, Hospices Civils de Lyon, 69437 Lyon, France; genevieve.lina-granade@chu-lyon.fr; 4 Constitutional Genetics Unit, Versailles Hospital, 78150 Versailles, France; gdelplancq@ght78sud.fr; 5 Inserm U1245, Department of Genetics and Reference Center for Developmental Disorders, Normandie University, University of Rouen Normandy, Rouen University Hospital (CHU Rouen), 76031 Rouen, France; anne-marie.guerrot@chu-rouen.fr; 6 Department of Medical Genetics, Tours University Hospital (CHRU Tours), 37044 Tours, France; annick.toutain@univ-tours.fr; 7 Department of Otorhinolaryngology, Otoneurology and Pediatric Otorhinolaryngology, University of Toulouse, Purpan University Hospital (CHU Purpan), 31300 Toulouse, France; dupin-deguine.d@chu-toulouse.fr; 8 Department of Medical Genetics, Bordeaux University Hospital (CHU de Bordeaux), 33076 Bordeaux, France; marine.legendre@chu-bordeaux.fr; 9 Inserm, CTM UMR1231, GAD Team, FHU TRANSLAD, Department of Genetics, Reference Center for Developmental Anomalies and Congenital Malformation Syndromes, Université Bourgogne, Dijon University Hospital (CHU Dijon Bourgogne), 21079 Dijon, France; estelle.colin@chu-dijon.fr; 10 Department of Pediatric Otorhinolaryngology and Head and Neck Surgery, Necker–Enfants Malades Hospital, Assistance Publique–Hôpitaux de Paris (AP-HP), Paris Cité University, 75015 Paris, France; natalie.loundon@aphp.fr; 11 Genetics of Rare Ophthalmological, Auditory and Mitochondrial Disorders, Inserm UMR_S1163, Imagine Institute, 75015 Paris, France

**Keywords:** *COCH*, DFNA9, hearing loss, genotype–phenotype, vestibular dysfunction, novel variant, autosomal dominant NSHL

## Abstract

**Objectives:** To characterize heterozygous pathogenic *COCH* variants in a French cohort with non-syndromic sensorineural hearing loss (NSHL) and assess genotype–phenotype correlations in autosomal dominant NSHL (DFNA9). **Setting:** National Reference Center for Genetic Hearing Loss, Necker–Enfants Malades Hospital, Paris, France. **Methods:** This retrospective observational study included 69 individuals from 20 unrelated families diagnosed with DFNA9 (2005–2025). All individuals underwent clinical and audiological evaluations and genetic testing via targeted *COCH* Sanger sequencing or next-generation sequencing (NGS) panels. Variants were interpreted according to ACMG guidelines. Audiometric profiles and vestibular data were collected. **Results:** Seven known pathogenic *COCH* variants were found in ten families, and ten novel likely pathogenic variants in the others. Variants in vWFA domains were associated with early or late onset, progressive, bilateral and symmetrical hearing loss. Three variants (p.Gln410Arg, p.Ile450Val, p.Cys542Arg) were associated with congenital or prelingual onset, an atypical DFNA9 presentation. Variants in the LCCL domain were associated with later-onset hearing loss and more frequent vestibular dysfunction. Vestibular abnormalities were observed in about half of early-onset cases. **Conclusions:** *COCH*-related hearing loss is a rare cause of autosomal dominant NSHL, with only 20 families identified over two decades within the French network. This study expands the mutational spectrum of *COCH* by reporting ten novel variants and supports a domain-specific genotype–phenotype correlation. These findings improve the understanding of DFNA9 variability and have direct implications for clinical diagnosis, prognosis, and genetic counseling.

## 1. Introduction

Hearing loss (HL) is the most common sensory deficit worldwide. Currently more than 1.5 billion people (nearly 20% of the global population) live with hearing loss and 430 million of them have disabling hearing loss. It is expected that by 2050, there could be over 700 million people with disabling hearing loss [1]. Its prevalence increases with age, affecting more than 25% of people over 60 years old [1]. In France, recent epidemiological data estimate that 25% of adults are affected by HL, with approximately 4% experiencing disabling hearing impairment [2].

Hearing loss may result from environmental or genetic factors. Sixty to 80% of prelingual HL cases have a genetic origin [3]. It is estimated that genetic factors account for approximately 25% of sensorineural hearing loss in young adults [4]. To date, more than 150 genes have been associated with non-syndromic hearing loss (NSHL), among which over 60 follow an autosomal dominant inheritance pattern (ADNSHL) [3,5]. One of the genes implicated in ADNSHL is *COCH* (coagulation factor C homolog), located on chromosome 14q12–q13. Pathogenic heterozygous variants in *COCH* cause DFNA9, characterized by progressive postlingual sensorineural HL, often associated with vestibular dysfunction including recurrent vertigo [6].

*COCH* encodes cochlin, composed of 550 amino acids and is the most abundant protein in the extracellular matrix of the inner ear, particularly in the spiral ligament and spiral limbus [7]. Cochlin contains several structural domains, including a signal peptide (SP), an LCCL domain (limulus factor C, cochlin, and late gestation lung protein), and two von Willebrand factor A-like (vWFA) domains. Most reported pathogenic *COCH* variants are missense variations affecting either the LCCL or vWFA domains, which are critical for protein structure, secretion, and function [7,8].

Pathogenic variants in *COCH*, lead to protein misfolding and aggregation which impair intracellular trafficking. These alterations interfere with cochlin secretion and cleavage, potentially causing progressive damage to cochlear and vestibular structures [8,9,10]. While several *COCH* pathogenic variants have been described in European populations, including a well-known Dutch founder variant (c.151C>T; p.Pro51Ser) [11], the prevalence of *COCH*-related HL remains poorly identified in other populations, and genotype–phenotype correlations are not yet fully established except for the Dutch recurrent variation [12].

In this study, we report a cohort of 69 individuals from 20 unrelated French families carrying heterozygous pathogenic or likely pathogenic *COCH* variants. We describe ten novel variants and analyze the audiovestibular associated phenotypes, aiming to refine the DFNA9 genotype–phenotype correlations and provide insights to improve diagnosis and genetic counseling.

## 2. Materials and Methods

### 2.1. Subjects

For all individuals in our cohort (69), aged from 9 months to over 60 years, audiometric and, when available, vestibular test results were collected. The severity of the hearing impairment was defined following BIAP (International Bureau for Audiophonology) recommendations [13].

We included 20 families through the French Reference Center for Genetic Hearing Loss between 2005 and 2025. All probands (20) presented with non-syndromic sensorineural hearing loss (NSHL) and were found to carry a heterozygous pathogenic or likely pathogenic *COCH* variant (classified as ACMG class 4 or 5).

### 2.2. Molecular Analysis

Genetic testing for *COCH* variants was performed using different molecular approaches:

In probands, next-generation sequencing (NGS) was carried out with a targeted hearing loss gene panel comprising either 110 or 220 genes. The complete list of genes included in each panel is provided in the Supplementary Table S1. For some probands from the earliest families included in the study, *COCH* analysis was performed by direct Sanger sequencing of the gene, as gene panel testing was not available at that time.

In available relatives, variant segregation was assessed by Sanger sequencing.

DNA extraction was performed on whole blood samples using different DNA extraction kits, according to their respective manufacturers’ instructions, after obtaining written informed consent from all participants or their legal guardians.

NGS was performed using a custom hearing loss gene panel (Twist Bioscience, South San Francisco, CA, USA) on an Illumina NextSeq 500 platform (Illumina, San Diego, CA, USA), following the manufacturer’s instructions. Variant analysis and filtering were conducted using Polyweb, a custom interface developed by the University of Paris Cité Bioinformatics Platform. Rare variants (frequency < 1% in dbSNP, 1000 Genomes, and gnomAD) with predicted moderate or high impact (including frameshift, splice-site, start/stop codon, missense variants, and in-frame indels) were retained for further analysis. Variants previously classified as benign or likely benign (ClinVar or internal database) were excluded.

For *COCH*, variants were retained if they met the following criteria:

- Observed with fewer than 5 alleles in gnomAD;
- Predicted to be pathogenic by at least one in silico tool among CADD (>20), PolyPhen-2, REVEL, AlphaMissense, or SpliceAI (score ≥ 0.10);
- Not retained if only a low CADD score (<22) or only “possibly damaging” prediction by PolyPhen-2 was present, and no stronger evidence supported pathogenicity;
- No other more plausible genetic cause of hearing loss was identified.

All prioritized variants were visually inspected using the Integrative Genomics Viewer (IGV, Broad Institute, Cambridge, MA, USA; version 2.16.0) to confirm read alignment and variant quality.

Sanger sequencing of the *COCH* coding region was performed in selected affected and unaffected relatives to assess segregation, according to Applied Biosystem chemistry guide (Thermo Fischer scientific, Waltham, MA, USA).

## 3. Results

### 3.1. Clinical Findings

Among the 69 individuals of our cohort, 65 were symptomatic at the time of evaluation, while four were asymptomatic carriers including two pediatric individuals. Audiograms were available for 38 patients; for the others, clinical data were obtained during clinical interview. The age of onset of HL was available for 48 individuals in our cohort.

The cohort included 33 males and 36 females. In all cases, hearing loss (HL) was sensorineural, bilateral, progressive and predominantly symmetrical (symmetrical in 26 patients, asymmetrical in 13 patients and lack of information in 29 patients). The audiometric profiles were mainly down-sloping. The severity of HL ranged from mild to profound at the time of analysis.

Based on the reported age of onset, when available, individuals were categorized into two groups:

- Early-onset HL (≤25 years): 21 individuals from eight families. Among them, six patients presented congenital or clearly prelingual HL, a phenotype rarely associated with COCH variations to date (five). Notably, one of these patients carried two pathogenic compound heterozygous variants in the GJB2 gene, which is sufficient to account for congenital hearing loss; this individual was therefore excluded from the analyses.
- Late-onset HL (>25 years): 27 individuals from 13 families, with the average age at diagnosis between 40 and 50 years.

One family (family 13) was found in both groups with ages at diagnosis ranging from 12 to 40 years but predominantly 12 years (2 individuals/3).

Regarding the two pediatric asymptomatic individuals carrying COCH heterozygous variation, audiological data were normal at the age of 9 months and 15 months respectively.

Vestibular function was assessed in 19 individuals. Dysfunction was present in 14 individuals and was more frequently observed in late-onset cases (12/14) than in early-onset patients (2/14 patients). Vertigo and/or tinnitus were reported inconsistently (in 16 of 65 symptomatic individuals), confirming that vestibular signs may be absent or subtle in some cases [3]. Only one patient with normal vestibular function had tinnitus. No dizziness was present in patients with normal vestibular function.

Age at independent walking was available in 23 patients and was within the normal range in all cases, suggesting preserved early motor development, even in patients with congenital HL.

### 3.2. Genetic Findings

Seventeen distinct heterozygous variants in the COCH gene were identified in our cohort:

- Previously reported pathogenic variants (*n* = 7): p.Pro51Ser, p.Ala76Thr, p.Gly88Glu, p.Ile109Thr, p.Phe121Ser, p.Ile372Thr, and p.Cys542Arg found in 10 families. The p. Ala76Thr variation was found in two unrelated families and the p.Ile109Thr variation was identified in three unrelated families.
- Likely pathogenic, previously unreported variants (*n* = 10): p.Ala68Gly, p.Asn182Asp, p.Gly276Cys, p.Gly293Valfs*6, p.Cys351Gly, p.Cys358Tyr, p.Asn367Lys, p.Gln410Arg, p.Ile437Ser and p.Ile450Val, each found in one independent family.

Variants located in the vWFA domains seemed to be predominantly associated with early-onset HL (<25 years) (7/11 variants), including the three variants found in congenital or prelingual cases (p. Gln410Arg, p.Ile450Val, p.Cys542Arg). In contrast, five of the nine variants associated with late-onset HL (>25 years), were located in the LCCL domain, in accordance with previous reports linking LCCL domain variants to vestibular dysfunction and adult-onset hearing loss [7,8].

Four variants (p. Gly276Cys, p.Gly293Valfs*6, p.Cys358Tyr and p.Ile437Ser) were located in the vWFA domains and, according to our classification (early-onset before age 25 and late onset after age 25), were associated with a late onset. These families may present analysis biases:

- The family, 11, carrying the p.Gly276Cys variant includes four affected patients diagnosed at ages 33 (1), 50 (2), and 55 (1).
- The family, 12, carrying the p. Gly293Valfs*6 variant includes two affected patients, diagnosed at ages 29 and 40 respectively.
- The family, 14, carrying the p. Cys358Tyr variant has only one patient whose HL was diagnosed at age 43 and the family with the p.Ile437Ser variant includes four affected patients, with the age at diagnosis being 40 for one patient and unavailable for the other three. For all these individuals, the age of onset may be much earlier than the age of diagnosis.

**Figure 1:**
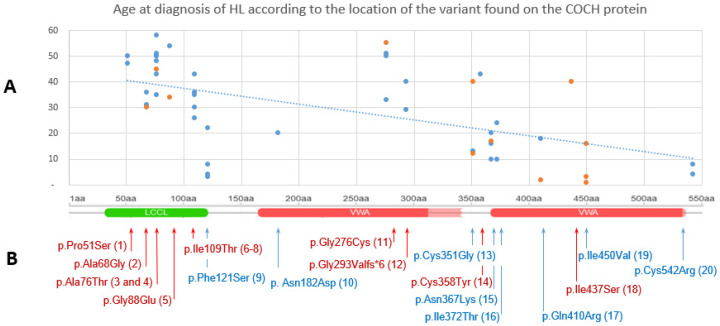
Distribution of the age at diagnosis of HL in our cohort according to the location of the variant found on the COCH protein. (**A**) The trend curve shows a decrease in the age at diagnosis for patients carrying variants located in the vWA domains. The blue dots indicate patients for whom audiograms were available And the orange dots indicate patients for whom audiograms were not available. (**B**) Localisation of the *COCH* variants on the protein. LCCL (Limulus factor C, Cochlin, and late gestation lung protein) domain is shown in green and vWA (von Willebrand factor A-like) domains in red. Blue arrows show variations associated with early-onset HL (≤25 years) while red ones show variations associated with late onset HL (>25 years).

To explore potential genotype–phenotype correlations, we analyzed the distribution of age at diagnosis of hearing loss according to the location of the variant within the COCH protein (Figure 1A). Variants located in the vWFA domains were associated with both early- and late-onset hearing loss, whereas variants in the LCCL domain were predominantly associated with later-onset hearing loss. Notably, the trend line indicates a tendency toward a lower age at diagnosis for variants located within the vWFA domains (Figure 1B).

Table 1 summarizes the clinical features and molecular findings of the families in our cohort, providing an overview of genotype–phenotype correlations.

**Figure 2:**
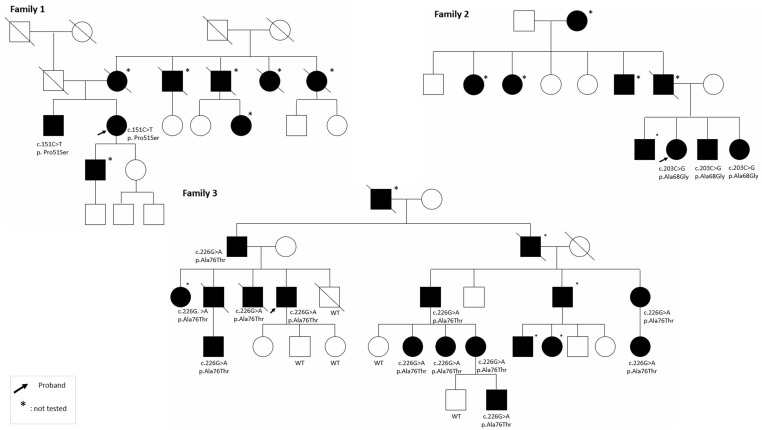

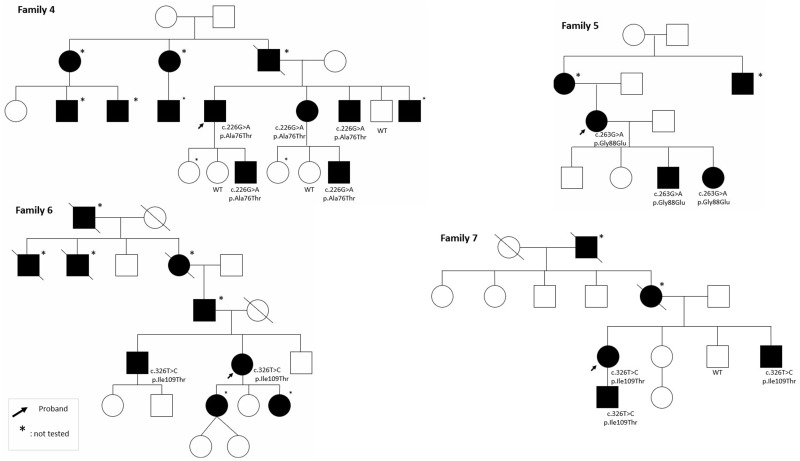

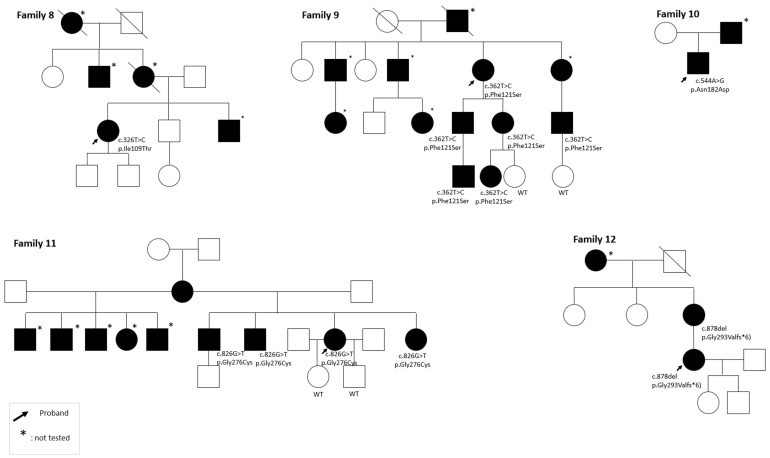

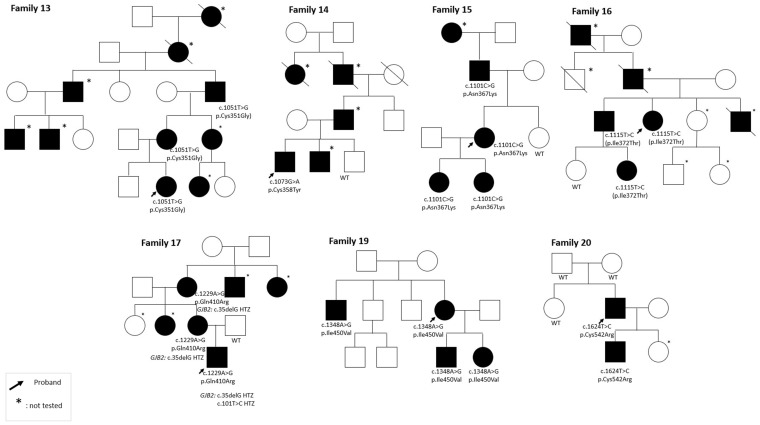
Pedigrees of families 1 to 17, 19 and 20 and molecular results. One pedigree was excluded from the figure (family 18) to comply with ethical and confidentiality requirements; this exclusion did not affect the overall analysis. Squares represent males, circles represent females, filled symbols indicate affected individuals, and slashed symbols indicate deceased individuals.

Figure 2 presents the pedigrees of the families included in our cohort. These family trees illustrate the segregation of *COCH* variants, and the inheritance patterns observed across affected and unaffected individuals. They provide a visual overview of intrafamilial phenotypic variability and support the assessment of genotype–phenotype correlations.

Supplementary Table S2, presents individual-level clinical and molecular data for affected patients whenever available.

## 4. Discussion

This study provides new insights into *COCH*-related hearing loss (DFNA9) by reporting a large French cohort with ten novel likely pathogenic variants. Although DFNA9 remains a rare cause of autosomal dominant non-syndromic hearing loss (ADNSHL), our findings underscore its clinical heterogeneity, including early-onset and even congenital forms of hearing loss, which are not traditionally associated with *COCH* mutations.

Typically, DFNA9 is described as a postlingual, progressive disorder with onset in the third to sixth decade of life, often associated with vestibular dysfunction such as imbalance or vertigo [6]. However, our data challenge this traditional model. We report three families with prelingual or congenital hearing loss associated with variants located in the vWFA2 domain (p.Gln410Arg, p.Ile450Val, and p.Cys542Arg). Similar early-onset presentations, in the second decade, have been reported only sporadically for patients with variants in the vWFA2 domain [6,14,15], suggesting they may be underdiagnosed, particularly in pediatric populations lacking vestibular complaints or family history.

In addition, one family carrying the p.Phe121Ser variant exhibits clearly early-onset hearing loss, beginning between 8 and 22 years of age, and even prelingual in two individuals. Unexpectedly, this variant is located in the terminal part of the LCCL domain, suggesting the likely involvement of additional factors influencing the phenotype.

Our results support and refine previously suggested domain-specific genotype–phenotype correlations in COCH-related HL [6,14]. Most variants in the LCCL domain, such as p.Pro51Ser and p.Ala76Thr, were associated with later-onset HL and a higher prevalence of vestibular symptoms. This domain is critical for cochlin cleavage and secretion, and disrupting these processes has been shown to lead to accumulation of uncleaved protein and matrix aggregation, contributing to vestibular pathology [14,16].

Conversely, variants in the vWFA domains notably vWFA2 were previously associated with earlier-onset HL, including congenital or prelingual forms, as seen in our cohort. These domains contribute to structural protein stability and extracellular interactions. Missense variations in this region may induce intracellular misfolding, aberrant multimerization, or toxic aggregation, disrupting cochlear cell function at an early stage.

This domain-specific variability suggests different underlying mechanisms: cochlin retention and intracellular stress in early-onset vWFA cases, versus extracellular matrix remodeling and fibrosis in LCCL-associated vestibular disease. A significant limitation of this hypothesis could be the increasing early access of patients to clinical diagnosis and genetic testing.

Despite shared genotypes within families, our cohort showed considerable intrafamilial phenotypic variability in age of onset and severity. This observation aligns with prior studies and points toward possible age-dependent penetrance, modifying genes, or environmental factors modulating the phenotype [14].

In Family 17, the interpretation of the pathogenic role of the c.1229A>G (p. Gln410Arg) variant in *COCH* remains challenging. Although the proband, her mother, and her maternal grandmother all carry this variant, the proband presents with prelingual hearing loss in a context of compound heterozygosity for two pathogenic *GJB2* variants (c.35delG and c.101T>C), a well-established cause of congenital and early-onset hearing loss. This genetic background precludes attributing the early-onset of hearing loss in the proband to the *COCH* variant. Notably, the maternal grandmother, who carries only a single heterozygous *GJB2* c.35delG variant, also reported congenital hearing loss, suggesting the possible involvement of additional genetic or non-genetic factors. The mother, who carries the *COCH* variant with a monoallelic *GJB2* variation (c.35delG), developed hearing loss during adolescence, a clinical course more consistent with previously reported *COCH*-associated phenotypes. Taken together, these findings suggest that the c.1229A>G (p.Gln410Arg) variant in *COCH* may contribute to hearing loss with variable expressivity or age of onset, while early-onset phenotypes in this family are likely influenced by other genetic factors, particularly *GJB2*. Another case of probable other causative or environmental factors is one individual reported in the literature, with a mutation in the LCCL domain, c.266C>A; Pro89His, displaying unilateral congenital hearing loss [15].

Vestibular dysfunction was more frequent in late-onset cases and those with LCCL variants, in accordance with previous findings [6,12,14]. However, its absence in early-onset cases does not exclude DFNA9, as vestibular hypofunction may remain subclinical for years. Subtle vestibular signs may be missed in absence of formal testing.

These results highlight the need for systematic vestibular evaluations, both for diagnosis and longitudinal follow-up.

The molecular mechanisms underlying DFNA9-associated hearing loss have been extensively investigated and support a predominantly dominant-negative or toxic gain-of-function effect of mutant cochlin rather than simple haploinsufficiency [1,2,3]. Previous functional studies have shown that pathogenic missense variants, particularly those located within the LCCL and vWFA domains, may impair proper protein folding, intracellular trafficking, and secretion, resulting in intracellular retention, abnormal cochlin aggregation, and extracellular deposition within the inner ear matrix [2,4]. These alterations are thought to disrupt extracellular matrix homeostasis and progressively impair cochlear and vestibular function [3,5]. In this context, the novel variants identified in our cohort are predicted to affect highly conserved residues and are likely to alter cochlin structure and stability, consistent with established pathogenic mechanisms reported for COCH-related hearing loss. Variants affecting the LCCL domain may interfere with proteolytic processing and secretion, whereas those located in the vWFA domains could disrupt calcium-dependent structural interactions required for proper extracellular matrix assembly and intermolecular interactions [6,7]. In silico predictions together with segregation data and their localization within known mutational hotspots strongly support their deleterious effect and classification as likely pathogenic, although functional validation will be required to confirm these predicted molecular consequences.

Our findings argue for the inclusion of *COCH* in routine diagnostic gene panels for both typical adult-onset ADNSHL and early-onset symmetrical SNHL, particularly when GJB2 and other common genes are excluded. The low number of identified DFNA9 families over two decades underscores the rarity of *COCH*-related HL but also reflects potential under-recognition of atypical phenotypes.

Because of variable expressivity, genetic counseling must be cautious: asymptomatic carriers may remain so for decades, while others may develop profound HL or disabling vestibular dysfunction. This supports individualized follow-up strategies, including audiological and vestibular monitoring, for asymptomatic carriers and pediatric patients. Although the age of onset of HL can vary between families and within the same family, penetrance appears to be complete between 50 and 55 years of age.

Moreover, the identification of ten novel variants, absent or rare in gnomAD and predicted pathogenic by multiple tools, underlines the challenges of variant interpretation in rare hearing losses. Population databases such as GnomAD, All Of Us or deCAF may present interpretation biases for late postlingual hearing loss. Functional studies are often lacking, and interpretation must integrate clinical, segregation, and bioinformatic evidence. Data sharing in public databases such as ClinVar is crucial to improve global variant classification efforts.

Limitations of this study include its retrospective nature, incomplete clinical data, and absence of functional assays to validate the pathogenicity of novel variants. While segregation and in silico data provide strong support, experimental confirmation (e.g., immunofluorescence, cochlin secretion assays) would strengthen causal links.

Future directions include:

- Functional validation of novel *COCH* variants;
- Longitudinal vestibular assessments in carriers, especially in pediatric cases;
- *COCH* analysis in NGS panels even in case of early hearing loss onset;
- Investigation of modifier genes or regulatory elements influencing phenotypic expression.

A better understanding of COCH pathophysiology could pave the way for targeted therapeutic approaches, which are currently being explored for other forms of genetic hearing loss.

Recently, antisense oligonucleotides (ASOs) or gene therapy have been developed to selectively target the mutant *COCH* allele, thereby preventing its translation into protein without affecting the wild-type allele. By inhibiting the production of toxic cochlin proteins, these ASOs allow the exclusive expression of the normal protein. When delivered to the inner ear, this strategy has the potential to slow or even halt the progression of hearing loss in individuals with DFNA9, offering a promising novel therapeutic option [17].

## 5. Conclusions

In this study, we report a multicenter French cohort of 69 individuals from 20 unrelated families carrying heterozygous pathogenic or likely pathogenic variants in *COCH* gene. Our results expand the mutational spectrum of DFNA9 by identifying ten novel variants and highlight a broader phenotypic variability than previously recognized.

Notably, several families presented with early-onset hearing loss, including congenital or prelingual onset, challenging the classical view of DFNA9 as an exclusively adult-onset disorder. Our analysis supports a domain-specific genotype–phenotype correlation, with variants in the vWFA domains associated with earlier-onset hearing loss and those in the LCCL domain more frequently linked to vestibular dysfunction and later onset.

These findings underline the importance of including *COCH* in diagnostic hearing loss gene panels, even in early-onset or atypical cases, and emphasize the need for systematic audiovestibular assessment in carriers. The marked intrafamilial variability further highlights the importance of individualized genetic counseling and longitudinal clinical monitoring.

Future studies incorporating functional analyses, larger cohorts, and long-term follow-up will be essential to fully understand the mechanisms underlying *COCH*-related disease and to guide the development of targeted therapeutic strategies for patients with DFNA9.

## Figures and Tables

**Table 1:** Clinical and molecular characteristics by *COCH* variant.

Family	Nucleotidic Variant	Protein Variant	Exon	Domain	N Patients	Age of Onset [Range] (N)	HL Caracteristic	HL Severity	Progression	Vestibular Dysfunction	Previously Reported or Novel	Notes/Extra Findings
1	c.151C>T	p.Pro51Ser	4	LCCL	2	50	bilateral symetric	profound	progressive	bilateral areflexia	HGMD Pro	Classic DFNA9 phenotype
2	c.203C>G	p.Ala68Gly	4	LCCL	3	32	bilateral	NA	NA	bilateral areflexia	Novel Variant	
3	c.226G>A	p.Ala76Thr	4	LCCL	12	48	bilateral, asymetric(5) symetric(2) et NA(5)	severe –profound	progressive	hypo or areflexia	HGMD Pro	Classic DFNA9 phenotype
4	c.226G>A	p.Ala76Thr	4	LCCL	5	46	bilateral, asymetric(5) symetric(2) and NA(5)	mild-severe	progressive	NA	HGMD Pro	Classic DFNA9 phenotype
5	c.263G>A	p.Gly88Glu	5	LCCL	3	44	bilateral	mild	progressive	NA	HGMD Pro	tinnitus associated in 1 patient
6	c.326T>C	p.Ile109Thr	5	LCCL	2	39	bilateral symetric(2) asymetric(2) NA(2)	severe	stable(1) progressive(3) NA(2)	areflexia	HGMD Pro	tinnitus and vertigo associated
7	c.326T>C	p.Ile109Thr	5	LCCL	3	30		profound		hyporeflexia	HGMD Pro	tinnitus and vertigo associated
8	c.326T>C	p.Ile109Thr	5	LCCL	1	30		severe		fluctuating	HGMD Pro	tinnitus and vertigo associated
9	c.362T>C	p.Phe121Ser	5	LCCL	6	early [8–22] prelingual(2)	bilateral symetric	slight-mild to profound	quickly progressive skislope	anormal vestibular function (2) normal(1) NA(2)	HGMD Pro	tinnitus and vertigo inconstant Early-onset despite LCCL location
10	c.544A>G	p.Asn182Asp	8	vWFA1	1	Early [20]	bilateral symetric	slight	NA	NA	novel Variant	tinnitus
11	c.826G>T	p.Gly276Cys	10	vWFA1	4	~30	bilateral symetric or asymetric	severe	progressive	Mild dysfunction	novel variant	
12	c.878del	p.Gly293Valfs*6	10	vWFA1	2	[29-40]	bilateral symetric	slight-mild	progressive	normal(1) NA(1)	novel variant	Frameshift
13	c.1051T>G	p.Cys351Gly	11	between vWFA1 and vWFA2	3	probably early(teenage(1) 12(1) 40(1)	bilateral symetric	mild	progressive	NA	novel variant	tinnitus(1) no tinnitus and vertigo(1) NA(1) Novel variant
14	c.1073G>A	p.Cys358Tyr	11	between vWFA1 and vWFA2	1	43	asymetric	mild	progressive	abnormal	novel variant	left vestibular schwanoma associated
15	c.1101C>G	p.Asn367Lys	11	vWFA2	4	early [10–24]	bilateral symetric	severe	quickly progressive ski slope	NA	novel variant	tinnitus in 1 patient
16	c.1115T>C	p.Ile372Thr	11	vWFA2	3	early [10–20]	bilateral symetric(2) asymetric(1)	mild-severe	progressive	NA	HGMD Pro	
17	c.1229A>G	p.Gln410Arg	11	vWFA2	3	very early: congenital (1, HTZ GJB2) prelingual(1, HTZ composite GJB2)	bilateral symetric(3)	mild to Severe	progressive (1) stable(1) NA(1)	Normal (1) NA(2)	novel variant	composite *GJB2* variants present in 1 patient
18	c.1310T>G	p.Ile437Ser	11	vWFA2	5	40	bilateral	NA	progressive	NA	novel variant	
19	c.1348A>G	p.Ile450Val	11	vWFA2	4	very early: Congenital (1) prelingual (1) NA(2)	bilateral symetric(1) asymetric(2) NA(1)	slight-mild	stable(2) NA(2)	Normal	novel variant	vestibular dysfunction(1) NA(3)
20	c.1624T>C	p.Cys542Arg	11	vWFA2	2	early (before 4 to 8)	bilateral symetric	slight	progressive	NA	HGMD Pro	reported 2 times in literature

## Data Availability

The original contributions presented in this study are included in the article/Supplementary Material. Further inquiries can be directed to the corresponding author(s).

## Supplementary Materials

The following supporting information can be downloaded at: https://www.mdpi.com/article/10.3390/genes17050588/s1: Table S1: List of hearing loss genes panel; Table S2: Individual-level clinical and molecular data for affected patients whenever available.

## Abbreviations

The following abbreviations are used in this manuscript:

HL	hearing loss
NSHL	non-syndromic hearing loss
ADNSHL	autosomal dominant NSHL
DFNA	deafness autosomal dominant
COCH	coagulation factor c homology
DFNB	deafness autosomal recessive
MIM	Mendelian inheritance in man number
